# Association of Vascular Risk Factors and Cerebrovascular Pathology With Alzheimer Disease Pathologic Changes in Individuals Without Dementia

**DOI:** 10.1212/WNL.0000000000209801

**Published:** 2024-09-17

**Authors:** Luigi Lorenzini, Alessio Maranzano, Silvia Ingala, Lyduine E. Collij, Mario Tranfa, Kaj Blennow, Carol Di Perri, Christopher Foley, Nick C. Fox, Giovanni B. Frisoni, Sven Haller, Pablo Martinez-Lage, Daisy Mollison, John O'Brien, Pierre Payoux, Craig Ritchie, Philip Scheltens, Adam J. Schwarz, Carole H. Sudre, Betty M. Tijms, Federico Verde, Nicola Ticozzi, Vincenzo Silani, Pieter Jelle Visser, Adam Waldman, Robin Wolz, Gael Chételat, Michael Ewers, Alle Meije Wink, Henk Mutsaerts, Juan Domingo Gispert, Joanna M. Wardlaw, Frederik Barkhof

**Affiliations:** From the Department of Radiology and Nuclear Medicine (L.L., S.I., L.E.C., M.T., A.M.W., F.B.), Amsterdam University Medical Centre, Vrije Universiteit; Amsterdam Neuroscience (L.L., S.I., L.E.C., A.M.W., H.M.), Brain Imaging, Amsterdam, The Netherlands; Department of Neurology and Laboratory of Neuroscience (A.M., F.V., N.T., V.S.), IRCCS Istituto Auxologico Italiano, Milan, Italy; Department of Radiology (S.I.), Copenhagen University Hospital Rigshospitalet; Cerebriu A/S (S.I.), Copenhagen, Denmark; Clinical Memory Research Unit (L.E.C.), Department of Clinical Sciences, Lund University, Malmö, Sweden; Department of Advanced Biomedical Sciences (M.T.), University “Federico II,” Naples, Italy; Department of Psychiatry and Neurochemistry (K.B., C.H.S.), Institute of Neuroscience and Physiology, the Sahlgrenska Academy at the University of Gothenburgn; Clinical Neurochemistry Laboratory (K.B.), Sahlgrenska University Hospital, Mölndal, Sweden; Neuroradiology Department (C.D.P.), University Hospital of Coventry and Warwickshire (UHCW), Coventry; GE HealthCare (C.F.), Amersham; Dementia Research Centre (N.C.F.), UCL Queen Square Institute of Neurology; UK Dementia Research Institute at University College London (N.C.F.), United Kingdom; Laboratory Alzheimer's Neuroimaging and Epidemiology (G.B.F.), IRCCS Istituto Centro San Giovanni di Dio Fatebenefratelli, Brescia, Italy; University Hospitals and University of Geneva (G.B.F.); CIMC - Centre d’Imagerie Médicale de Cornavin (S.H.), Place de Cornavin 18, Genève, Switzerland; Department of Surgical Sciences (S.H.), Radiology, Uppsala University, Sweden; Department of Radiology (S.H.), Beijing Tiantan Hospital, Capital Medical University, P. R. China; Centro de Investigación y Terapias Avanzadas (P.M.-L.), Neurología, CITA‐Alzheimer Foundation, San Sebastián, Spain; Centre for Clinical Brain Sciences (D.M., A.W., J.M.W.), The University of Edinburgh; Department of Psychiatry (J.O.B.), School of Clinical Medicine, CB2 0SP, University of Cambridge, United Kingdom; Department of Nuclear Medicine (P.P.), Toulouse University Hospital; ToNIC (P.P.), Toulouse NeuroImaging Center, University of Toulouse, Inserm, UPS, France; Edinburgh Dementia Prevention (C.R.), Centre for Clinical Brain Sciences, Outpatient Department 2, Western General Hospital, University of Edinburgh Brain Health Scotland (C.R.), Edinburgh, United Kingdom; Alzheimer Center Amsterdam (P.S., B.M.T., P.J.V.), Neurology, Vrije Universiteit Amsterdam, Amsterdam UMC location VUmc; Amsterdam Neuroscience (P.S., B.M.T., P.J.V.), Neurodegeneration, Amsterdam, The Netherlands; Takeda Pharmaceuticals Ltd. (A.J.S.), Cambridge, MA; Department of Medical Physics and Biomedical Engineering (C.H.S.), Centre for Medical Image Computing (CMIC), University College London (UCL); MRC Unit for Lifelong Health & Ageing at UCL (C.H.S.), University College London; School of Biomedical Engineering and Imaging Sciences (C.H.S.), King's College London, United Kingdom; Department of Pathophysiology and Transplantation (F.V., N.T., V.S.), “Dino Ferrari” Center, Università degli Studi di Milano, Milan, Italy; Alzheimer Center Limburg (P.J.V.), Department of Psychiatry and Neuropsychology, School of Mental Health and Neuroscience, 6229 GS, Maastricht University, The Netherlands; Division of Neurogeriatrics (P.J.V.), Department of Neurobiology, Care Sciences and Society, Karolinska Institutet, Stockholm, Sweden; Department of Medicine (A.W.), Imperial College London; IXICO (R.W.), EC1A 9PN, London, United Kingdom; Université de Normandie (G.C.), Unicaen, Inserm, U1237, PhIND “Physiopathology and Imaging of Neurological Disorders”, institut Blood-and-Brain @ Caen-Normandie, Cyceron, Caen, France; German Center for Neurodegenerative Diseases (DZNE) (M.E.), Munich, Germany; Ghent Institute for Functional and Metabolic Imaging (GIfMI) (H.M.), Ghent University, Belgium; Barcelonaβeta Brain Research Center (BBRC) (J.D.G.), Pasqual Maragall Foundation; CIBER Bioingeniería (J.D.G.), Biomateriales y Nanomedicina (CIBER-BBN), Madrid; IMIM (Hospital del Mar Medical Research Institute) (J.D.G.); Universitat Pompeu Fabra (J.D.G.), Barcelona, Spain; UK Dementia Research Institute Centre at the University of Edinburgh (J.M.W.); and Institutes of Neurology and Healthcare Engineering (F.B.), University College London, United Kingdom.

## Abstract

**Background and Objectives:**

Vascular risk factors (VRFs) and cerebral small vessel disease (cSVD) are common in patients with Alzheimer disease (AD). It remains unclear whether this coexistence reflects shared risk factors or a mechanistic relationship and whether vascular and amyloid pathologies have independent or synergistic influence on subsequent AD pathophysiology in preclinical stages. We investigated links between VRFs, cSVD, and amyloid levels (Aβ_1-42_) and their combined effect on downstream AD biomarkers, that is, CSF hyperphosphorylated tau (P-tau_181_), atrophy, and cognition.

**Methods:**

This retrospective study included nondemented participants (Clinical Dementia Rating < 1) from the European Prevention of Alzheimer's Dementia (EPAD) cohort and assessed VRFs with the Framingham risk score (FRS) and cSVD features on MRI using visual scales and white matter hyperintensity volumes. After preliminary linear analysis, we used structural equation modeling (SEM) to create a “cSVD severity” latent variable and assess the direct and indirect effects of FRS and cSVD severity on Aβ_1-42_, P-tau_181_, gray matter volume (baseline and longitudinal), and cognitive performance (baseline and longitudinal).

**Results:**

A total cohort of 1,592 participants were evaluated (mean age = 65.5 ± 7.4 years; 56.16% F). We observed positive associations between FRS and all cSVD features (all *p* < 0.05) and a negative association between FRS and Aβ_1-42_ (β = −0.04 ± 0.01). All cSVD features were negatively associated with CSF Aβ_1-42_ (all *p* < 0.05). Using SEM, the cSVD severity fully mediated the association between FRS and CSF Aβ_1-42_ (indirect effect: β = −0.03 ± 0.01), also when omitting vascular amyloid-related markers. We observed a significant indirect effect of cSVD severity on P-tau_181_ (indirect effect: β = 0.12 ± 0.03), baseline and longitudinal gray matter volume (indirect effect: β = −0.10 ± 0.03; β = −0.12 ± 0.05), and baseline cognitive performance (indirect effect: β = −0.16 ± 0.03) through CSF Aβ_1-42_.

**Discussion:**

In a large nondemented population, our findings suggest that cSVD is a mediator of the relationship between VRFs and CSF Aβ_1-42_ and affects downstream neurodegeneration and cognitive impairment. We provide evidence of VRFs indirectly affecting the pathogenesis of AD, highlighting the importance of considering cSVD burden in memory clinics for AD risk evaluation and as an early window for intervention. These results stress the role of VRFs and cerebrovascular pathology as key biomarkers for accurate design of anti-amyloid clinical trials and offer new perspectives for patient stratification.

## Introduction

There is a frequent co-occurrence of vascular risk factors (VRFs), such as hypertension, obesity, and diabetes mellitus, with Alzheimer disease (AD) pathology, including amyloid-β (Aβ) and hyperphosphorylated tau (P-tau_181_) deposition, brain atrophy, and cognitive decline.^1^ Furthermore, recent studies have shown associations between AD pathology and cerebral small vessel disease (cSVD),^2^ especially white matter hyperintensities (WMHs),^3^ and less consistently with perivascular spaces (PVSs)^4^ and cerebral microbleeds (CMBs).^5^ However, it remains unclear whether this association is merely the result of shared risk factors or whether VRFs and cSVD directly relate to amyloid metabolism and clearance, representing an integral component of early AD pathophysiology. Understanding the role of cSVD in the pathogenesis of AD is key for clinical practice because it would provide a biomarker to guide interventional programs targeting the management of VRFs in individuals at risk of dementia. In addition, stratification of individuals based on cerebrovascular load might optimize selection strategies for enrollment in AD clinical trials targeting amyloid.

Conflicting evidence on the synergistic effect of both VRFs and cSVD with amyloid deposition on subsequent AD pathologic events has been reported. Two recent studies showed that VRFs^6^ and WMHs^7^ enhanced tau deposition and hippocampal neurodegeneration, respectively, in amyloid-positive individuals. However, other studies found cSVD and amyloid deposition to be 2 independent, but additive, mechanisms contributing to the appearance of cognitive symptoms in aging individuals.^8^ While these preliminary findings imply that VRFs and cSVD are related to amyloid burden and downstream effects, few studies have considered both cSVD and VRFs together in the same models^9^ (eTable 1) and most have limited their analyses to single radiologic indices of cSVD. As such, it remains unclear whether these vascular components and amyloid deposition act independently or interact with each other to promote tau accumulation, neurodegeneration, and eventually cognitive decline.

In this work, we explored the relationship between VRF markers, (composite markers of) cSVD severity (accounting for the distinct effects of arteriolosclerosis and CAA-related imaging markers), CSF AD biomarkers (Aβ_1-42_, P-tau_181_), and cognitive performance in individuals without dementia from the European Prevention of Alzheimer's Dementia (EPAD) cohort. Using structural equation modeling (SEM), we examined the direct and indirect contributions of VRFs and cSVD to the amyloid pathologic cascade and cognitive performance. We hypothesize that, in individuals without dementia, cardiovascular and cerebrovascular factors may contribute to amyloid deposition and related events, including p-tau deposition, neurodegeneration, and cognitive impairment.

## Methods

### Study Participants

Data for this study were drawn from the latest data release of the EPAD (ep-ad.org) cohort.^10^ EPAD participants were preselected from existing population-based cohorts and contacted by the EPAD sites in case of absence of disorders that could interfere with trial participation, absence of dementia, and openness to potentially participate in intervention studies. Potential participants were then invited for a screening/baseline visit, after which EPAD eligibility was confirmed if they met the following criteria: age older than 50 years; no diagnosis of dementia, that is, Clinical Dementia Rating (CDR) scale < 1; and absence of any major cerebrovascular pathologic signs (such as large infarct in the territory of main arteries) that may affect cognition in the opinion of the neuroradiologist (mentioned further).^11,12^ Further EPAD recruitment information can be found in previous publications.^12^ During the same visit, all participants underwent CSF and MRI acquisitions, while a subset of participants had longitudinal MRI and cognitive evaluation data available. All participants provided written informed consent.

### Vascular Risk Score

Individual vascular risk was computed using the Framingham risk score (FRS), a semiquantitative composite algorithm based on modifiable and nonmodifiable cardiovascular risk factors. The score, stratified by sex, was calculated by assigning a targeted amount of points for each preset range of age (expressed in years), total and HDL cholesterol levels, systolic blood pressure values (keeping into account the assumption of antihypertensive medication), and diabetes and smoking status.^13^ In the absence of blood measures, self-reported hypercholesterolemia was used to score cholesterol as described in previous studies.^14^

### Cognitive Testing

The EPAD neuropsychological battery data were collected with standardized procedures and included the Mini-Mental State Examination (MMSE),^15^ the CDR Scale,^16^ and the Repeatable Battery for the Assessment of Neuropsychological Status (RBANS) scores.^17^ Rates of change (ROC) of total RBANS scores were computed in participants with longitudinal data available as the difference in the score between the last and the first visit, divided by the years passed.

### CSF Analysis

CSF biomarkers were quantified using a harmonized preanalytical protocol, and analyses were centrally performed with the fully automatized Roche Elecsys System at the Clinical Neurochemistry Laboratory, Mölndal, Sweden.^11^ Concentrations of Aβ_1-42_ and P-tau_181_ were determined according to the manufacturer's instructions. Participants were classified as amyloid-positive (A+) or amyloid-negative (A-) using cutoff values, previously validated in the same cohort,^18,19^ of ≤1,000 pg/mL for Aβ_1-42_ positivity and of ≥27 pg/mL for P-tau_181_ (T- or T+) and further assigned to AT stages.^20^

### MRI Acquisition and Processing

The brain MRI protocol was harmonized across sites and included 3D T1-weighted (3D T1w), 3D fluid-attenuated inversion recovery (FLAIR), 2D T2w, and 2D T2* or SWI images.^21^ FreeSurfer 7.0.1 was used to derive volumes of a previously defined AD-signature mask—including temporal pole, inferior and middle temporal, inferior and superior parietal, precuneus, posterior cingulate, and entorhinal cortex volume—from both baseline and follow-up 3D T1w images.^22^ Atrophy ROC were computed in participants with longitudinal MRI data available as the difference in the volume between the last and the first visit, divided by the years passed. WMH volumes were computed from FLAIR images using the Bayesian model selection toolbox (BaMoS).^23^ Regional values of WMHs were obtained by averaging lesions within atlas regions taking into account lobar boundaries (frontal, parietal, temporal, and occipital) and distance between the ventricular surface and cortex (periventricular and deep), resulting in a total of 8 WMH regional values, and normalized for total white matter volume.

### Radiologic Assessment of Cerebrovascular Pathology

Visual MRI reads were centrally performed (by 3 trained readers blinded to participants' characteristics) according to the STandards for ReportIng Vascular changes on nEuroimaging (STRIVE) criteria.^24^ Visual rating included the following categorical scales: a 0–4 scale for PVS in the basal ganglia (BG) and centrum semiovale (CS); the Fazekas scale (0–3 each) for periventricular (PVH) and deep WMH (DWMH); presence (yes/no) of deep CMBs; presence of more than 2 lobar CMBs; and lacunes (0, 1, 2, >2). A more detailed description of the scores can be found in eMethods.

### Statistical Analysis

All analyses were conducted in R version 4.0.3 (r-project.org/). Data normalization strategies and analysis steps are described in eMethods and eFigure 1. Generally, the performed analysis followed 2 steps described as follows: descriptive analysis and structural equation modeling.

#### Descriptive Analysis

In the descriptive analysis, we assessed the preliminary association between vascular risk factors, cSVD indices, and AD CSF biomarkers.

##### Association of Vascular Risk Factors (FRS) With cSVD and AD CSF Biomarkers

The association between FRS (independent variable) and cSVD features (dependent variables) was assessed using separate generalized linear models, correcting for sex and APOE status (ε4carrier/noncarrier). Linear regression models were used for continuous outcomes (WMH volumes); logistic regression models were used for binary outcomes (lobar and deep CMBs); and multinomial logistic regression models were used for ordinal outcomes (Fazekas deep and periventricular scores, lacunes, and PVS, as described above). Model *p* values used for the 8 WMH regions of interest were adjusted for multiple comparisons. Linear models were then used to investigate the effect of FRS on CSF Aβ_1-42_ and P-tau_181_, separately. The same models were rerun using the FRS computed without age and adjusting for age.

##### Association of cSVD With AD CSF Biomarkers

Next, we investigated the relationship of each of our cSVD indices with CSF Aβ_1-42_ and P-tau_181_ using linear models. When predicting P-tau_181_, an interaction term between candidate cSVD variables and Aβ_1-42_ was included to assess possible interaction effects of cerebrovascular and amyloid pathology. All models were corrected for age, sex, and APOE ε4 status (carrier/noncarrier). Linear models used for the 8 WMH regions of interest were adjusted for multiple comparisons using Bonferroni correction.

#### Structural Equation Modeling

To determine how VRFs and cSVD interact with AD biomarkers, we used structural equation modeling (SEM, eMethods). SEM was performed in R using the “lavaan” package (version 0.6–12). Within the SEM framework, we created a “cSVD severity” latent variable to capture a comprehensive (latent) dimension driving cerebrovascular health, using confirmatory factor analysis of radiologic indices of cSVD^9,25,26^: Fazekas PVH and DWMH scores, PVS in the BG and CS, deep and lobar CMBs, and lacunes. We then used this latent factor in a preliminary mediation analysis and in a full model, described as follows.

##### Mediation Analysis

**Figure 1:**
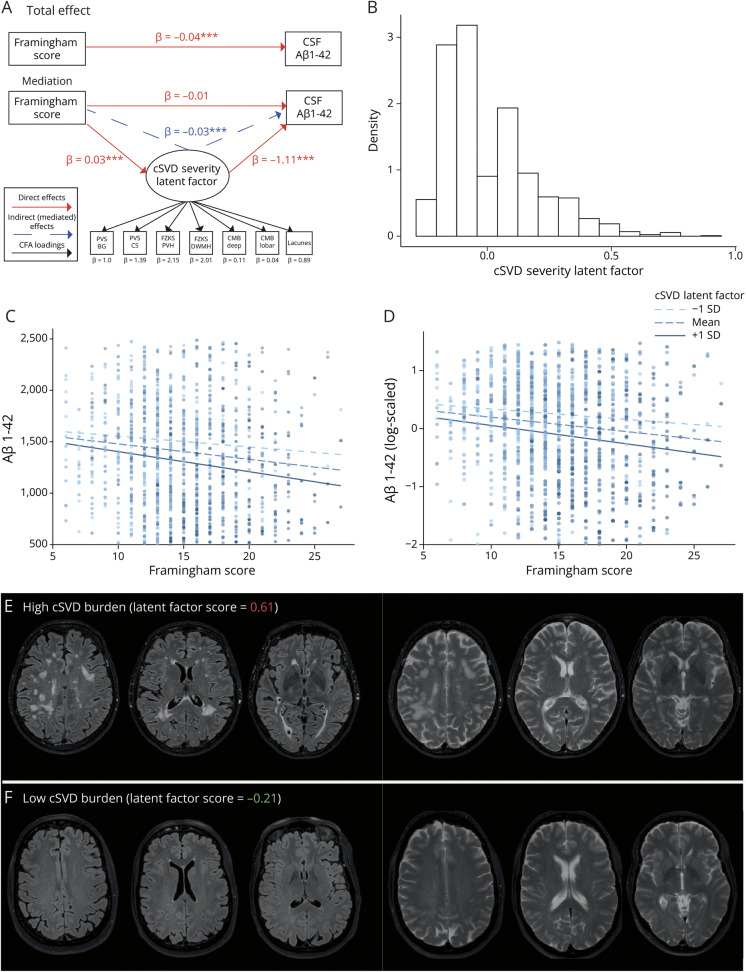
Mediation Analysis and cSVD Burden Latent Factor (A) Illustration of the mediation analysis, including total and mediated effects. After SEM convention, observed variables are depicted as rectangles and latent variables as ovals. Red arrows are used to illustrate direct effects. Blue arrows are used to illustrate indirect (mediated) effects. Black arrows are used to illustrate confirmatory factor analysis (CFA) loadings. *p < 0.05, **p < 0.01, and ***p < 0.001. (B) Distribution (histogram) of the estimated cSVD latent factor through CFA. (C) Association between Framingham scores and raw CSF Aβ_1-42_ values stratified by scores at the cSVD latent factor. (D) Association between Framingham scores and log-scaled CSF Aβ_1-42_ values stratified by scores at the cSVD latent factor. (E) Illustration of FLAIR and T2w scans from a participant with a high score in the cSVD latent factor. (F) Illustration of FLAIR and T2w scans from a participant with a low score in the cSVD latent factor. CMB = cerebral microbleed; cSVD = cerebral small vessel disease; D = deep; FZKS DWMH = Fazekas deep white matter hyperintensity; PV = periventricular; FZKS PVH = Fazekas periventricular hyperintensity; PVS-BG = perivascular spaces in basal ganglia; PVS-CS = perivascular spaces in centrum semiovale.

As a preliminary step, we built a model to test the mediation effect of the cSVD severity in the association between FRS and Aβ_1-42_ (Figure 1A).

##### Full Model

We then used SEM to model the relationship between 5 observed variables (z-scored): FRS, CSF Aβ_1-42_, CSF P-tau_181_, gray matter volume in the AD-signature mask, and cognitive performance as quantified using the global RBANS score (mentioned further) and the “cSVD severity” latent variable.

**Figure 2:**
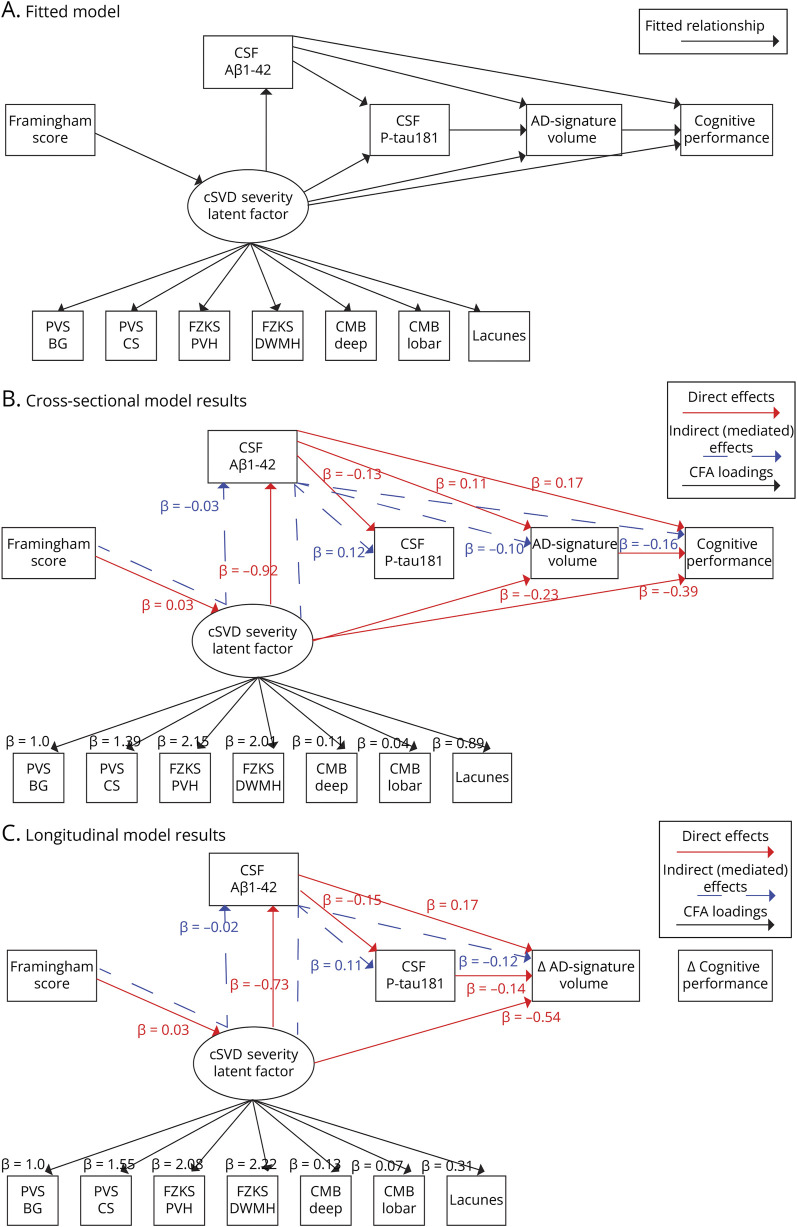
Structural Equation Model After SEM convention, observed variables are depicted as rectangles and latent variables as ovals. Arrows are used to illustrate relations included in the model. (A) The structural equation model fitted to the data after model selection. (B) The significant (*p* < 0.05) paths and standardized coefficients of the model fitted on cross-sectional data. (C) The significant (*p* < 0.05) paths and standardized coefficients of the model fitted using rates of change of gray matter atrophy and cognitive performance. Direct effects are shown in red, and indirect effects are shown in dotted blue arrows. All model coefficients are listed in eTables 17 and 18 of supplementary materials. AD = Alzheimer disease; CMB = cerebral microbleed; cSVD = cerebral small vessel disease; FZKS DWMH = Fazekas deep white matter hyperintensity; FZKS PVH = Fazekas periventricular hyperintensity; PVS-BG = perivascular spaces in basal ganglia; PVS-CS = perivascular spaces in centrum semiovale; ROC = rates of change.

A model selection step was used to find the best model to describe the influence of vascular factors on the amyloid cascade of events (eMethods). The selected model is shown in Figure 2 and included a direct relationship of FRS with the cSVD severity and of the latter with Aβ_1-42_; the effect of both Aβ_1-42_ and cSVD severity on P-tau_181_, gray matter volume, and cognitive performance; the effect of P-tau_181_ on gray matter volume; and effect of gray matter volume on cognitive performance. In addition to these direct effects, the model was also used to estimate the indirect (mediating) effect of amyloid on the relationship between cSVD severity and downstream AD events (P-tau_181_, gray matter volumes, and cognitive performance). The same model was then fit in the subset of participants having longitudinal MRI and cognitive evaluation data, using ROC of gray matter volume in the AD-signature mask and cognitive performance. As for the linear models, all the relationships in the model were corrected for age, sex, and APOE ε4 status (carrier/noncarrier).

##### Sensitivity Analysis

Sensitivity analyses were performed to evaluate the effect of specific covariates and stratifications and are reported in eMethods. These included evaluation of single FRS item effect on cSVD indices and CSF scores, stratified for sex, APOE ε4 status (carrier/noncarrier), and CDR. Moreover, to disentangle the effect of cerebral amyloid angiopathy (CAA) and arteriolosclerosis on AD biomarkers, we performed the SEM without including both lobar CMB and PVS-CS in the “cSVD severity” latent factor, according to the recent Boston criteria 2.0.^27^

### Data Availability

EPAD data can be accessed on request on the EPAD website: ep-ad.org/open-access-data/overview/.

## Results

### Cohort Characteristics

A total number of 1,718 participants, who had performed both MRI and CSF analysis, were initially evaluated for this study. In line with previous studies,^28,29^ and to specifically address AD-related pathologic changes,^30,31^ we excluded 126 participants with suspected non-Alzheimer pathology, that is, A-T+ (eMethods). The final sample consisted of 1,592 participants. A subset of 460 participants had longitudinal MRI and cognitive evaluation data available with an average follow-up time of 16 months.

The whole cohort characteristics (n = 1,592) across the study population and according to the AT status are provided in Table 1 and eTable 2. The mean age was 65.5 (±7.4) years, 894 (56.2%) were women, and 423 (26.6%) had a CDR = 0.5. Overall, participants had a low-intermediate cardiovascular and cerebrovascular burden, and higher FRS and cSVD radiologic scores were observed in more advanced AT stages. Characteristics of excluded A-T+ participants (n = 126) are listed in eTables 3 and 4. Characteristics of the subset of participants having longitudinal MRI and cognitive evaluation data are presented in eTable 5. An overview of all the performed analysis steps is given in eTable 6.

**Table 1:** Participant Characteristics

	A-T- N = 1,029		A+T- N = 397		A+T+ N = 166		Whole cohort N = 1,592	
Age; mean (SD)		64.3 ± 7.2		66.6 ± 7.4		70.1 ± 6.4		65.5 ± 7.4
Sex = male; N (%)		430 (41.8)		186 (46.9)		82 (49.4%)		698 (43.2%)
Years of education; mean (SD)		14.5 ± 3.6		14.6 ± 3.8		13.5 ± 4.0		14.5 ± 3.7
MMSE; mean (SD)		28.8 ± 1.4		28.3 ± 1.89		26.5 ± 2.9		28.4 ± 1.9
CDR = 0.5; N (%)		187 (18.2)		127 (32.0)		109 (65.7%)		423 (26.6%)
APOE status; N (%)	N = 1,008		N = 390		N = 163			
E2|E2		6 (0.6)		0 (0.0)		0 (0.0%)		6 (0.4%)
E2|E3		120 (11.7)		23 (5.8)		5 (3.0%)		148 (9.3%)
E2|E4		25 (2.4)		12 (3.0)		3 (1.8%)		40 (2.5%)
E3|E3		591 (57.4)		154 (38.8)		41 (24.7%)		786 (49.4%)
E3|E4		253 (24.6)		166 (41.8)		86(51.8%)		505 (31.7%)
E4|E4		13 (1.3)		35 (8.8)		28 (16.9%)		76 (4.8%)
FRS; mean (SD)	N = 1,029	14.2 ± 4.1	N = 397	15.4 ± 3.8	N = 166	16.5 ± 4.2	N = 1,319	14.8 ± 4.1
PVS-BG; N (%)	N = 1,014		N = 391		N = 162		N = 1,567	
0		46 (4.5)		8 (2.0)		4 (2.5%)		58,217.7 (3.7%)
1		843 (83.1)		316 (80.8)		120 (74.1%)		1,279 (81.6%)
2		108 (10.7)		48 (12.3)		24 (14.8%)		180 (11.5%)
3		16 (1.6)		17 (4.3)		11 (6.8%)		44 (2.8%)
4		1 (0.1)		2 (0.5)		3 (1.9%)		6 (0.4%)
PVS-CS; N (%)	N = 1,014		N = 391		N = 162		N = 1,567	
0		111 (10.9)		35 (9.0)		8 (4.9%)		154 (9.8%)
1		591 (58.3)		195 (49.9)		68 (42.0%)		854 (54.5%)
2		231 (22.8)		113(28.9)		48 (29.6%)		392 (25.0%)
3		72 (7.1)		42 (10.7)		33 (20.4%)		147 (9.4%)
4		9 (0.9)		6 (1.5)		5 (3.1%)		20 (1.3%)
Fazekas DWMH; N (%)	N = 1,014		N = 391		N = 162		N = 1,567	
0		366 (36.1)		105 (26.9)		34 (21.0%)		505 (32.2%)
1		515 (50.8)		205 (52.4)		71 (43.8%)		791 (50.5%)
2		123 (12.1)		65 (16.6)		49 (30.2%)		237 (15.1%)
3		10 (1.0)		16 (4.1)		8 (4.9%)		34 (2.2%)
Fazekas PVH; N (%)	N = 1,014		N = 391		N = 162	58 (35.8%)	N = 1,567	
0		644 (63.5)		196 (50.1)		61 (37.7%)		898 (57.3%)
1		281 (27.7)		141 (36.1)		39 (24.1%)		483 (30.8%)
2		81 (8.0)		50 (12.8)		4 (2.5%)		170 (10.8%)
3		8 (0.8)		4 (1.0)				16 (1.0%)
CMB lobar; N (%)	N = 1,029		N = 397		N = 166		N = 1,592	
<2		1,011 (98.3)		383 (96.5)		150 (90.4%)		1,544 (97%-0%)
≥2		18 (1.7)		14 (3.5)		16 (9.6%)		48 (3.0%)
CMB deep; N (%)	N = 1,029		N = 397		N = 166		N = 1,592	
<1		1,026 (99.7)		394 (99.2)		162 (97.6%)		1,582 (99.4%)
≥1		3 (0.3)		3 (0.8)		4 (2.4%)		10 (0.6%)
Lacunes; N (%)	N = 1,020		N = 393		N = 163		N = 1,576	
0		946 (92.7)		343 (87.3)		149 (91.4%)		1,438 (91.2%)
1		54 (5.3)		32 (8.1)		7 (4.3%)		93 (5.9%)
2		14 (1.4)		8 (2.0)		4 (2.5%)		26 (1.6)
>2		6 (0.6)		10 (2.5)		3 (1.8%)		19 (1.2)
WMH volume; mean (SD)	N = 689	4,734.7 ± 6,632.2	N = 279	7,389.53 ± 9,823.2	N = 114	8,713.29 ± 10099.3	N = 082	5,838 ± 8,108

Abbreviations: CDR = Clinical Dementia Rating; CMB = cerebral microbleed, cSVD = cerebral small vessel disease, DWMH = deep white matter hyperintensity; FRS = Framingham risk score; MMSE = Mini-Mental State Examination; N= number; PVH = periventricular hyperintensity; PVS-BG = perivascular spaces in basal ganglia; PVS-CS = perivascular spaces in centrum semiovale; WMH = white matter hyperintensity.

### Descriptive Analysis

#### FRS Is Related to cSVD and AD CSF Markers

We observed a significant association between FRS and all cSVD features; model coefficients are listed in Table 2 and illustrated in eFigure 2. For multinomial logistic regression models, group contrasts are presented in eTable 7. Higher FRSs were significantly associated with higher PVS-BG and PVS-CS scores, higher Fazekas PVH and DWMH scores, and more lacunes. Higher FRSs were also observed in participants with ≥2 lobar CMBs and with at least 1 deep CMB. We further observed a positive association between FRSs and WMH volumes in all investigated regions, most strongly in frontal and parietal regions (eTable 8).

**Table 2:** Model Coefficients of Statistical Relationship of cSVD Indices With Framingham Risk Score (FRS)

	Framingham risk score			
PVS-BG	R^2^ = 0.21			
	Reference: PVS-BG = 0 (mean FRS = 12.80)			
	Mean FRS	OR	CI	*p* Value
1	14.41	1.08	1.01–1.17	0.026
2	16.46	1.27	1.16–1.38	<0.001
3	16.98	1.41	1.25–1.59	<0.001
4	18.33	1.55	1.18–2.01	0.001
PVS-CS	R^2^ = 0.22			
	Reference: PVS-CS = 0 (mean FRS = 12.53)			
	Mean FRS	OR	CI	*p* Value
1	14.46	1.14	1.08–1.21	<0.001
2	14.73	1.22	1.15–1.29	<0.001
3	16.99	1.38	1.28–1.48	<0.001
4	19.11	1.57	1.36–1.82	<0.001
FZKS DWMH	R^2^ = 0.21			
	Reference: FZKS DWMH = 0 (mean FRS = 13.12)			
	Mean FRS	OR	CI	*p* Value
1	14.77	1.11	1.07–1.15	<0.001
2	16.62	1.26	1.21–1.33	<0.001
3	16.95	1.43	1.27–1.59	<0.001
FZKS PVH	R^2^ = 0.22			
	Reference: FZKS PVH = 0 (mean FRS = 13.56)			
	Mean FRS	OR	CI	*p* Value
1	15.56	1.14	1.11–1.18	<0.001
2	17.06	1.29	1.22–1.35	<0.001
3	17.58	1.46	1.23–1.72	<0.001
CMB lobar	R^2^ = 0.22			
	Reference: CMB lobar <2 (mean FRS = 14.61)			
	Mean FRS	OR	CI	*p* Value
≥2	18.12	3.151	1.57–6.61	<0.001
CMB deep	R^2^ = 0.13			
	Reference: CMB deep <1 (mean FRS = 14.64)			
	Mean FRS	OR	CI	*p* Value
≥1	16.57	1.65	0.93–2.96	*p* = 0.024
Lacunes	R^2^ = 0.18			
	Reference: lacunes = 0 (mean FRS = 14.67)			
	Mean FRS	OR	CI	*p* Value
1	16.41	1.11	1.05–1.17	<0.001
2	16.61	1.19	1.05–1.34	0.003
>2	15.5	1.12	0.98–1.29	0.10

Abbreviations: CMB = cerebral microbleed; cSVD = cerebral small vessel disease; FZKS DWMH = Fazekas deep white matter hyperintensity; FZKS PVH = Fazekas periventricular hyperintensity; PVS-BG = perivascular spaces in basal ganglia; PVS-CS = perivascular spaces in centrum semiovale.

FRS was used as a predictor in multinomial or logistic regression models. For these models, odds ratios, confidence intervals (95%), and *p* values are reported. Odds ratios represent the increase (>1) in the risk of falling in a category (compared with the reference) for a 1-unit increase in the predictor variable. For multinomial models, goodness-of-fit metrics (McFadden R^2^) are reported. Coefficients refer to comparison with reference groups, and other group contrasts are listed in eTable 7.

Moreover, higher FRSs were significantly associated with lower CSF levels of Aβ_1-42_ (β = −0.04 ± 0.01; *p* < 0.001) and higher levels of P-tau_181_ (β = 0.05 ± 0.01; *p* < 0.001) (eFigure 3). The direction or the significance level of these analyses was not affected by correction of FRS for age (details not reported).

#### cSVD Is Associated With Aβ_1-42_ and P-tau_181_

**Figure 3:**
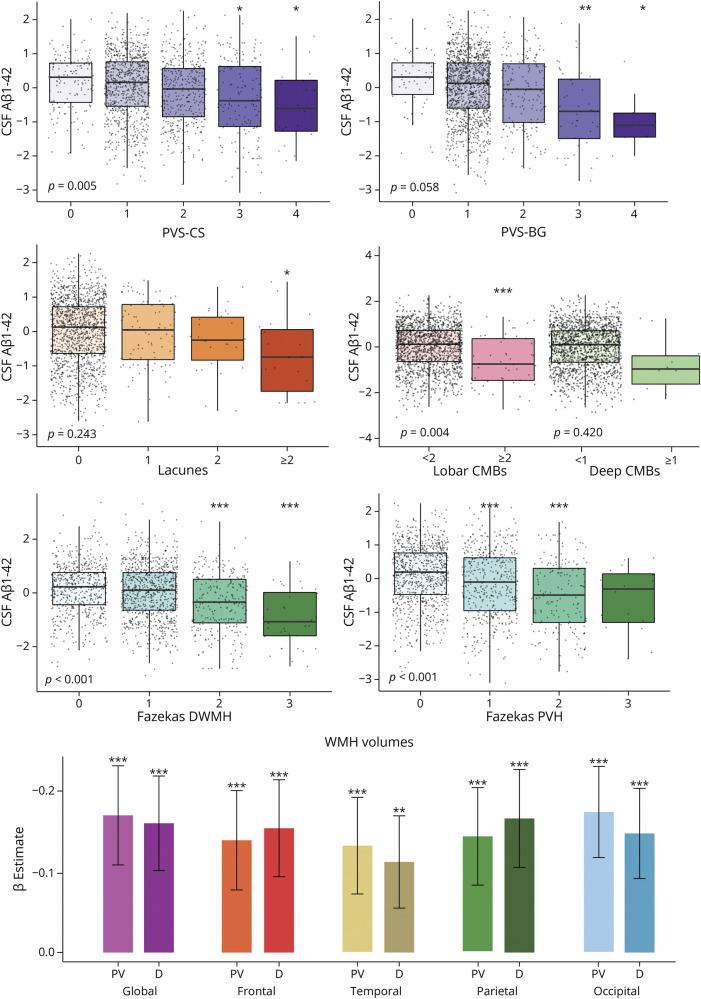
Association of CSF Aβ_1-42_ With cSVD Radiologic Scores Boxplot showing the association of CSF Aβ_1-42_ with PVS (BG and CS), lacunes, CMB (lobar and deep), and Fazekas scores (DWMH and PVH). *p* Values for the global association of cSVD scores and CSF Aβ_1-42_ are listed in the bottom left corner of boxplots. The association of CSF Aβ_1-42_ with WMH volumes is shown using barplots reporting the β coefficients and 95% confidence intervals of the models. Asterisks refer to significance levels as found in the multivariable general linear models correcting for age, sex, and APOE, compared with reference groups. Between-group comparisons are presented in eTable 9. CMB = cerebral microbleed; D = deep; DWMH = deep white matter hyperintensity; PV = periventricular; PVH = periventricular hyperintensity; PVS-BG = perivascular spaces in basal ganglia; PVS-CS = perivascular spaces in centrum semiovale. *p < 0.05, **p < 0.01, and ***p < 0.001.

Higher scores of all cSVD features were associated with lower CSF Aβ_1-42_ levels; model coefficients are listed in Table 3 and illustrated in Figure 3. Between-group estimated marginal mean contrasts are provided in eTable 9. Specifically, more abnormal (lower) CSF Aβ_1-42_ levels were associated with higher PVS-BG and PVS-CS scores, higher Fazekas PVH and DWMH scores, lobar (≥2) CMBs, but not deep (≥1) CMB, and >2 lacunes. Finally, larger WMH volumes of both deep and periventricular WMHs, globally and per lobe, were associated with lower CSF Aβ_1-42_ values (all *p* values <0.001) (Figure 3, eTable 10). Coefficients describing the relationship between cSVD indices and CSF P-tau_181_ are listed in eTable 11. Higher CSF P-tau_181_ levels were associated with higher PVS-CS and higher Fazekas PVH scores.

**Table 3:** Model Coefficients of Statistical Relationship of cSVD Indices With CSF Aβ_1-42_

	CSF Aβ_1-42_			
PVS-BG	R^2^ = 0.21			
	Reference: PVS-BG = 0 (mean Aβ_1-42_ = 1,490.85)			
	Mean Aβ_1-42_	β	Std. Error	*p* Value
1	1,355.86	−0.15	0.11	0.191
2	1,298.01	−0.13	0.13	0.336
3	1,006.18	−0.56	0.18	0.001
4	830.75	−0.85	0.38	0.022
PVS-CS	R^2^ = 0.21			
	Reference: PVS-CS = 0 (mean Aβ_1-42_ = 1,425.65)			
	Mean Aβ_1-42_	β	Std. Error	*p* Value
1	1,385.26	−0.05	0.08	0.451
2	11279.51	−0.12	0.08	0.155
3	1,221.18	−0.22	0.11	0.039
4	1,077.73	−0.47	0.21	0.026
FZKS DWMH	R^2^ = 0.23			
	Reference: FZKS DWMH = 0 (mean Aβ_1-42_ = 1,436.32)			
	Mean Aβ_1-42_	β	Std. Error	*p* Value
1	1,358.52	−0.08	0.05	0.12
2	1,157.82	−0.32	0.07	<0.001
3	857.64	−0.83	0.15	<0.001
FZKS PVH	R^2^ = 0.23			
	Reference: FZKS PVH = 0 (mean Aβ_1-42_ = 1,430.55)			
	Mean Aβ_1-42_	β	Std. Error	*p* Value
1	1,278.85	−0.18	0.05	<0.001
2	1,090.47	−0.44	0.07	<0.001
3	993.60	−0.40	0.23	0.07
CMB lobar	R^2^ = 0.20			
	Reference: CMB lobar <2 (mean Aβ_1-42_ = 1,351.59)			
	Mean Aβ_1-42_	β	Std. Error	*p* Value
≥2	1,011.36	−0.38	0.13	0.004
CMB deep	R^2^ = 0.20			
	Reference: CMB deep <1 (mean Aβ_1-42_ = 1,343.58)			
	Mean Aβ_1-42_	β	Std. Error	*p* Value
≥1	986.69	−0.09	0.11	0.423
Lacunes	R^2^ = 0.20			
	Reference: lacunes = 0 (mean Aβ_1-42_ = 1,353.70)			
	Mean Aβ_1-42_	β	Std. Error	*p* Value
1	1,310.70	0.01	0.09	0.946
2	1,148.59	−0.11	0.17	0.531
>2	973.46	−0.40	0.20	0.05

Abbreviations: CMB = cerebral microbleed; cSVD = cerebral small vessel disease; FZKS DWMH = Fazekas deep white matter hyperintensity; FZKS PVH = Fazekas periventricular hyperintensity; PVS-BG = perivascular spaces in basal ganglia; PVS-CS = perivascular spaces in centrum semiovale.

CSF Aβ_1-42_ was used as an outcome in linear models. For these models, beta coefficients, standard errors (Std. Errors), and *p* values are reported. For all models, goodness-of-fit metrics (Nagelkerke R^2^) are reported. Reported β-coefficients represent the change in Z-score of log-transformed values of the outcome variable (CSF Aβ_1-42_) for a 1-unit increase in the predictor variable. Coefficients refer to comparison with reference groups, and other group contrasts are listed in eTable 9.

#### cSVD Interacts With Aβ_1-42_ in Predicting P-tau_181_

We then explored whether the relationship between cSVD and p-tau181 was dependent on CSF Aβ_1-42_ (eFigure 4). We found a significant interaction between CSF Aβ_1-42_ and Fazekas scores, both PVH (R^2^ = 0.17, *p* < 0.001) and DWMH (R^2^ = 0.16, *p* < 0.001), in predicting P-tau_181_ levels. Specifically, a stronger negative association was found between CSF Aβ_1-42_ and CSF P-tau_181_ in participants with higher Fazekas PVH (coefficients per step: 0:β = 0.7 ± 0.03, *p* = 0.02; 1:β = −0.15 ± 0.04, *p* < 0.001; 2:β = −0.25 ± 0.06, *p* < 0.001; 3:β = −0.42 ± 0.26, *p* = 0.10) and DWMH (coefficients per step: 0:β = −0.03 ± 0.05, *p* = 0.57; 1:β = −0.02 ± 0.03, *p* = 0.62; 2:β = −0.28 ± 0.06, *p* < 0.001; 3:β = −0.14 ± 0.14, *p* = 0.30) scores. We further observed a significant interaction between CSF Aβ_1-42_ and lobar CMBs, with stronger negative association between CSF Aβ_1-42_ and CSF P-tau_181_ in participants with ≥2 lobar CMBs (<2: β = −0.05 ± 0.03, *p* = 0.07; ≥2: β = −0.34 ± 0.12, *p*=<0.001). No significant interaction between CSF Aβ_1-42_ and PVS nor lacunes on P-tau_181_ was found.

Similarly, WMH volumes globally (deep and periventricular) and in the parietal (deep and periventricular), occipital (deep), and temporal (periventricular) lobes showed significant interaction with CSF Aβ_1-42_ in predicting CSF P-tau_181_ (eTable 12). In all these regions, a higher WMH burden was related to a stronger negative relationship of CSF Aβ_1-42_ with P-tau_181_.

### Structural Equation Modeling

Positive significant (all *p* < 0.001) contributions (loadings) were observed for all cSVD indices used in the confirmatory factor analysis to identify the cSVD severity latent factor, with highest values for Fazekas scores, both PVH (β = 2.05 ± 0.12) and DWMH (β = 2.14 ± 0.13), followed by PVS, both in the BG (β = 1.0 ± 0.27) and CS (β = 1.46 ± 0.11), and also by the presence of lacunes (β = 0.86 ± 0.07) and of lobar (β = 0.09 ± 0.02) and deep (β = 0.03 ± 0.01) CMBs. The distribution of the cSVD severity latent factor is shown in Figure 1B. FLAIR and T2w scans for exemplar participants with low and high burden are shown in Figure 1E and F.

#### Mediation Analysis

While in the descriptive analysis we found that FRS was significantly associated with CSF Aβ_1-42_, the preliminary mediation analysis showed that this association was fully mediated by the cSVD severity (direct: β = −0.01, *p* = 0.34; indirect: β = −0.03, *p* < 0.001; and total: β = −0.04, *p* < 0.001). All coefficients are presented in Figure 1A and eTable 13. A graphical illustration of the indirect association is shown in Figure 1, C and D.

#### Full Model

In the selected SEM model (eTables 14 and 15, eFigures 5 and 6), we found a significant association of FRS with cSVD severity (β = −0.03 ± 0.01; *p* < 0.001) and a significant association of the latter with CSF Aβ_1-42_ (β = −0.92 ± 0.12; *p* < 0.001). CSF Aβ_1-42_ was significantly directly associated with CSF P-tau_181_ levels (β = −0.13 ± 0.03; *p* < 0.001), gray matter volumes (β = 0.11 ± 0.03; *p* < 0.001), and RBANS total scores (β = 0.17 ± 0.03; *p* < 0.001). cSVD severity was significantly directly associated with gray matter volumes (β = −0.23 ± 0.11; *p* = 0.002) and RBANS total scores (β = −0.39 ± 0.12; *p* < 0.001). Moreover, significant indirect effects of cSVD severity on P-tau_181_ (indirect effect: β = 0.12 ± 0.03; *p* < 0.001), gray matter volumes (indirect effect: β = −0.10 ± 0.03; *p* < 0.001), and RBANS total scores (indirect effect: β = −0.16 ± 0.03; *p* < 0.001) mediated by CSF Aβ_1-42_ were observed.

In the longitudinal model, comparable direct and indirect effects were observed on ROC of gray matter volumes (direct: β = −0.54 ± 0.22, *p* = 0.01; indirect: β = −0.12 ± 0.05, *p* = 0.02; total effect: β = −0.67 ± 0.22, *p* = 0.003) while no significant (direct or indirect) association was found between any included variable and ROC of RBANS total scores (eFigure 7). Model coefficients are illustrated in Figure 2 and listed in eTables 16–19. Additional results from sensitivity analyses and the effect of covariates in the SEM models are in line with the reported results and presented in eTables 20–23.

#### Disentangling Arteriolosclerosis and CAA

When excluding the lobar CMB and PVS-CS scores from the computation of cSVD severity latent factor to separate possible CAA-related effects, the results of the model remained unchanged (eTable 24).

## Discussion

This multicenter study has 3 main findings: first, we demonstrate the influence of VRFs and cSVD on CSF Aβ_1-42_ levels; second, we found an indirect association of VRFs with amyloid pathology through the presence of cSVD; and third, we showed that cSVD is indirectly associated with tau accumulation, gray matter atrophy (also over time), and cognitive impairment through amyloid pathology.

Our findings confirm previous studies that showed a relationship between WMHs and amyloid burden^3^ and further demonstrate that other cSVD radiologic markers—PVS, microbleeds, and lacunes—are also associated with amyloid pathology. Few studies have previously reported evidence on PVS, showing that higher burden of PVS in CS and BG was associated with higher levels of P-tau_181_, total tau, and neurogranin (a biomarker of synaptic degeneration) in amyloid-positive individuals.^4^ Another study used premortem and postmortem MRI to show increased amyloid accumulation in vessels with enlarged PVS.^32^ These results suggest that dysregulation of PVS function might result in impaired clearance mechanisms and facilitate amyloid deposition. In turn, CMBs and PVS are on one hand considered as a classic expression of vascular damage related to hypertensive vasculopathy, but also as a marker of CAA, depending on their location (deep vs lobar and BG vs CS).^5^ In this context, these radiologic findings could bring together the amyloid cascade and vascular AD hypothesis, as a downstream product of 2 separate pathways that facilitate subsequent neurodegeneration. In our models, arteriosclerotic markers were the main drivers, independent of vascular amyloid markers (lobar CMB and EPVS in CS).

In line with previous literature,^8^ our findings showed an association of VRFs with lower CSF Aβ_1-42_, reflecting Aβ deposition in the brain, and further suggest this effect to be mediated by the presence of brain vascular lesions. VRFs, such as hypertension, high cholesterol, and diabetes, can result in thickening of vessel walls, reduced vessel elasticity, and reduced vasoreactivity by alterations of the neurovascular unit, which not only reduce perfusion but also affect control of cerebral blood flow. Alongside inducing neurodegeneration directly (hit 1), the vascular dysfunction underlying cSVD could also indirectly enhance amyloid accumulation in the brain (hit 2).^33^ Specifically, impaired PVS function and vascular brain injury may lead to a failure in amyloid vascular clearance with the indirect consequence of reduced amyloid in CSF and elevated amyloid levels in the brain.^34^ It is important to note, however, that VRFs at midlife have the largest effect on brain health.^35^ Thus, the observed results could be partially obscured by the fact that we used FRS at older age to study this association.

Whether VRFs, amyloid, and cSVD act synergistically or independently in promoting the progression of AD is still a matter of debate.^36^ Using comprehensive models, we observed a stronger relationship between low CSF amyloid and tau pathology in the presence of several cSVD markers (interaction). SEM analysis demonstrated an association of cSVD severity with low CSF amyloid and how this can result in worse tau pathology, atrophy (also over time), and cognitive dysfunction (indirect effects), suggesting a synergistic contribution of these components. Of interest, findings from sensitivity analyses suggest that this contribution would be more pronounced in the early stages of the disease process (CDR = 0) or in the absence of a well-established risk factor of AD such as APOE ε4 allele (eMethods—sensitivity analyses), where the AD cascade might not be driven by amyloid and vascular risk factors might be more relevant. Previously, a higher VRF burden had been found to be significantly interacting with amyloid burden in predicting longitudinal brain atrophy and further cognitive decline.^6^ Likewise, other studies^37^ have shown that VRFs interact with subthreshold levels of amyloid Aβ, as detected by amyloid PET, to promote cognitive decline, by partially accelerating early tau accumulation, in a cohort of cognitively unimpaired individuals.

The collective evidence suggests a crucial involvement of cerebrovascular dysfunction in early AD-related biological events, with possible convergence of vascular and amyloid pathways to synergistically increase tau pathology, atrophy, and cognitive decline. Although this was not directly tested in this work, among several potential pathways, cSVD has been associated with inflammation in and around the perforating arterioles and capillaries and has been shown previously to relate to neuroinflammatory processes and astrogliosis.^38,39^ Such processes, in turn, have been proposed to mediate the relationship between amyloid and tau.^40^ cSVD-related neuroinflammatory processes may, therefore, constitute a co-pathology that triggers an increase in inflammatory markers and thus promote the amyloid-dependent pathologic processes, typical of AD.

In contrast to our results, recent studies suggested that, while both cSVD and amyloid pathology relate to faster atrophy rates and subsequent dementia, these effects seemed to be independent in the studied cohorts.^8^ The investigated disease stage might explain this paradoxical evidence. Indeed, our cohort consisted of nondemented participants, with almost one-third of them in the early biological stages of the Alzheimer continuum, when vascular insults might be expected to exert their strongest contributions on AD pathophysiology.^1^ Furthermore, the use of SEM allowed for a more detailed characterization of the relationship between several variables in a complex system, and SEM models might, therefore, be more sensitive than classical linear models used in previous studies. It is also important to note that previous studies proposed a bidirectional influence in the relationship between cSVD and amyloid, suggesting that amyloid deposition may in turn induce vasoconstriction and vessel wall damage, resulting in cSVD pathology and promoting circular feedback mechanisms.^41^ The selected model did not include a direct effect of amyloid on the cSVD severity latent factor, which may have been obscured by the relatively low overall amyloid burden of the studied cohort, resulting in limited power to detect this effect, or by the use of a global measure of amyloid that might be less sensitive compared with regional information, for example, using amyloid PET. Moreover, being mostly performed on cross-sectional data, our results do not provide evidence of any causal or directional mechanisms but simply advocate for a strong contribution of cSVD in the earliest stages of the disease. For example, alternative tested models (M1 and M3 in eFigure 5) have shown similar fit to the analyzed data, and therefore, different causal structures might also be considered. While we focused on the contribution of vascular pathology in the early AD stages, studies investigating the opposite amyloid-on-cSVD effect, or disentangling between the two, could benefit from including participants in more advanced disease stages, or affected by genetic AD variants where the effect of amyloid deposition on vascular components might be better discerned.^42^ In contrast to previous literature, we also did not find any effect on longitudinal cognitive performance. A possible explanation for this could be the short follow-up time that might not be enough to see significant cognitive changes in individuals without dementia. Furthermore, the investigated disease stage and the high frequency with which the cognitive tests were repeated (every 6 months, according to the EPAD study design) might have promoted some learning effects. Moreover, the scarcity of longitudinal data contained in EPAD did not allow for more sensitive modeling approaches often incorporated in SEM analysis, such as latent growth models.

Our work has several strengths, including the large number of predementia participants and the detailed radiologic evaluation. The large sample size allowed us to identify small, albeit significant, effects of FRS on CSF Aβ_1-42_, suggesting that even in individuals without dementia or individuals early along the AD continuum and with limited cardiovascular burden, the association is measurable and should be taken into account. However, we could not study causal mechanisms due to the nature of the study design. Future longitudinal studies, assessing midlife cardiovascular risk factors in healthy individuals, would be required to determine the causal relationship between vascular pathology and AD pathologic changes. The modifying effect of timely/effective treatment of midlife cardiovascular disease should be considered as an experimental manipulation. Repeated CSF samples and PET scans would be required for an accurate estimation of the onset of amyloid and tau pathology in individuals with high cardiovascular risk. Stratified analyses accounting for age, sex, APOE haplotype, level of neuroinflammation, glymphatic flow, sleep activity, and other relevant biological variables would be required to properly assess effect modifiers. Other limitations should also be noted. The absence of CSF Aβ_1-40_ did not allow us to measure the ratio between Aβ_1-42_ and Aβ_1-40_, which represents a more reliable biomarker of (nonvascular) amyloid pathology. Individuals with major cerebrovascular findings on MRI were excluded from EPAD; thus, some of the cSVD indices—such as microbleeds and lacunes—were underrepresented, and all SVD features were relatively mild. Considering the complexity of the investigated pathologic associations, other variables (here not considered) might also affect the observed relationships, such as diet, leisure activities, and depression. Other direct associations could also be considered in the future, such as the direct impact of cardiovascular factors on tau deposition, neurodegeneration, and cognitive impairment, independently of cSVD and Aβ_1-42_. In addition, the spatial distribution of tau has also been shown to carry important information about underlying etiologies^43^; future studies could use tau PET to better disentangle the effect of vascular factors on such pathologic mechanisms. It is important to note that the absence of neuropathologic data limited the assessment of independent contributions of CAA and arteriolosclerosis markers. However, in our sensitivity analysis, we showed that excluding lobar CMBs and PVS-CS, considered as CAA neuroradiologic indices in the most recent criteria,^27^ did not change the results of our main analysis. This suggests that arteriolosclerosis-related neuroradiologic abnormalities have a driving role in the observed associations, independently of CAA.

Taken together, our results highlight the important role of cSVD in early amyloid deposition and related events, including tau pathology and atrophy. Furthermore, the data suggest a route whereby VRFs can link through cSVD to promote the amyloid pathologic cascade of events in susceptible individuals. Overall, these findings suggest that VRFs and cSVD represent integral components of the early stages of the biological cascade that leads to neurodegeneration in AD and stress the importance of monitoring and controlling VRFs, not ignoring brain cSVD features, and accelerating the testing of agents that could improve the vascular dysfunction in cSVD as a way to help prevent development of AD.

## Appendix Authors

**Table:**

Name	Location	Contribution
Luigi Lorenzini, PhD	Department of Radiology and Nuclear Medicine, Amsterdam University Medical Centre, Vrije Universiteit; Amsterdam Neuroscience, Brain Imaging, The Netherlands	Drafting/revision of the manuscript for content, including medical writing for content; study concept or design; analysis or interpretation of data
Alessio Maranzano	Department of Neurology and Laboratory of Neuroscience, IRCCS Istituto Auxologico Italiano, Milan, Italy	Drafting/revision of the manuscript for content, including medical writing for content; analysis or interpretation of data
Silvia Ingala, MD, PhD	Department of Radiology and Nuclear Medicine, Amsterdam University Medical Centre, Vrije Universiteit; Amsterdam Neuroscience, Brain Imaging, The Netherlands; Department of Radiology, Copenhagen University Hospital Rigshospitalet; Cerebriu A/S, Copenhagen, Denmark	Study concept or design
Lyduine E. Collij, PhD	Department of Radiology and Nuclear Medicine, Amsterdam University Medical Centre, Vrije Universiteit; Amsterdam Neuroscience, Brain Imaging, The Netherlands; Clinical Memory Research Unit, Department of Clinical Sciences, Lund University, Malmö, Sweden	Drafting/revision of the manuscript for content, including medical writing for content
Mario Tranfa, MD	Department of Radiology and Nuclear Medicine, Amsterdam University Medical Centre, Vrije Universiteit, The Netherlands; Department of Advanced Biomedical Sciences, University “Federico II”, Naples, Italy	Drafting/revision of the manuscript for content, including medical writing for content
Kaj Blennow, MD, PhD	Department of Psychiatry and Neurochemistry, Institute of Neuroscience and Physiology, the Sahlgrenska Academy at the University of Gothenburg; Clinical Neurochemistry Laboratory, Sahlgrenska University Hospital, Mölndal, Sweden;	Major role in the acquisition of data
Carol Di Perri	University Hospital of Coventry and Warwickshire (UHCW), Neuroradiology Department, Coventry, United Kingdom	Major role in the acquisition of data
Christopher Foley, PhD	GE HealthCare, Amersham, United Kingdom	Major role in the acquisition of data
Nick C. Fox, MD	Dementia Research Centre, UCL Queen Square Institute of Neurology; UK Dementia Research Institute at University College London, United Kingdom	Major role in the acquisition of data
Giovanni B. Frisoni, MD	Laboratory Alzheimer’s Neuroimaging & Epidemiology, IRCCS Istituto Centro San Giovanni di Dio Fatebenefratelli, Brescia, Italy; University Hospitals and University of Geneva, Switzerland	Major role in the acquisition of data
Sven Haller, MD	CIMC - Centre d’Imagerie Médicale de Cornavin, Genève; Department of Surgical Sciences, Radiology, Uppsala University, Sweden; Department of Radiology, Beijing Tiantan Hospital, Capital Medical University, P. R. China	Major role in the acquisition of data
Pablo Martinez-Lage, MD, PhD	Centro de Investigación y Terapias Avanzadas, Neurología, CITA‐Alzheimer Foundation, San Sebastián, Spain	Major role in the acquisition of data
Daisy Mollison, MD	Centre for Clinical Brain Sciences, The University of Edinburgh, United Kingdom	Major role in the acquisition of data
John O′Brien, PhD	Department of Psychiatry, School of Clinical Medicine, University of Cambridge, United Kingdom	Major role in the acquisition of data
Pierre Payoux, MD	Department of Nuclear Medicine, Toulouse University Hospital; ToNIC, Toulouse NeuroImaging Center, University of Toulouse, 31300, Inserm, UPS, France	Drafting/revision of the manuscript for content, including medical writing for content
Craig Ritchie, PhD	Edinburgh Dementia Prevention, Centre for Clinical Brain Sciences, Outpatient Department 2, Western General Hospital, University of Edinburgh; Brain Health Scotland, Edinburgh, United Kingdom	Major role in the acquisition of data
Philip Scheltens, MD, PhD	Alzheimer Center Amsterdam, Neurology, Vrije Universiteit Amsterdam, Amsterdam UMC location VUmc; Amsterdam Neuroscience, Neurodegeneration, The Netherlands	Drafting/revision of the manuscript for content, including medical writing for content
Adam J. Schwarz, PhD	Takeda Pharmaceuticals Ltd., Cambridge, MA	Drafting/revision of the manuscript for content, including medical writing for content
Carole H. Sudre, PhD	Department of Psychiatry and Neurochemistry, Institute of Neuroscience and Physiology, the Sahlgrenska Academy at the University of Gothenburg, Sweden; Department of Medical Physics and Biomedical Engineering, Centre for Medical Image Computing (CMIC), University College London (UCL); MRC Unit for Lifelong Health & Ageing at UCL, University College London; School of Biomedical Engineering and Imaging Sciences, King’s College London, United Kingdom	Drafting/revision of the manuscript for content, including medical writing for content
Betty M. Tijms, PhD	Alzheimer Center Amsterdam, Neurology, Vrije Universiteit Amsterdam, Amsterdam UMC location VUmc; Amsterdam Neuroscience, Neurodegeneration, The Netherlands	Drafting/revision of the manuscript for content, including medical writing for content
Federico Verde, MD	Department of Neurology and Laboratory of Neuroscience, IRCCS Istituto Auxologico Italiano; Department of Pathophysiology and Transplantation, “Dino Ferrari” Center, Università degli Studi di Milano, Milan, Italy	Drafting/revision of the manuscript for content, including medical writing for content
Nicola Ticozzi, MD	Department of Neurology and Laboratory of Neuroscience, IRCCS Istituto Auxologico Italiano; Department of Pathophysiology and Transplantation, “Dino Ferrari” Center, Università degli Studi di Milano, Milan, Italy	Drafting/revision of the manuscript for content, including medical writing for content
Vincenzo Silani, MD	Department of Neurology and Laboratory of Neuroscience, IRCCS Istituto Auxologico Italiano; Department of Pathophysiology and Transplantation, “Dino Ferrari” Center, Università degli Studi di Milano, Milan, Italy	Drafting/revision of the manuscript for content, including medical writing for content
Pieter Jelle Visser, MD, PhD	Alzheimer Center Amsterdam, Neurology, Vrije Universiteit Amsterdam, Amsterdam UMC location VUmc; Amsterdam Neuroscience, Neurodegeneration; Alzheimer Center Limburg, Department of Psychiatry and Neuropsychology, School of Mental Health and Neuroscience, 6229 GS, Maastricht University, Maastricht, The Netherlands; Division of Neurogeriatrics, Department of Neurobiology, Care Sciences and Society, Karolinska Institutet, Stockholm, Sweden	Drafting/revision of the manuscript for content, including medical writing for content
Adam Waldman, MD, PhD	Centre for Clinical Brain Sciences, The University of Edinburgh; Department of Medicine, Imperial College London, United Kingdom	Major role in the acquisition of data
Robin Wolz, PhD	IXICO, EC1A 9PN, London, United Kingdom	Major role in the acquisition of data
Gael Chételat, PhD	Université de Normandie, Unicaen, Inserm, U1237, PhIND “Physiopathology and Imaging of Neurological Disorders”, institut Blood-and-Brain @ Caen-Normandie, Cyceron, France	Drafting/revision of the manuscript for content, including medical writing for content
Michael Ewers, PhD	German Center for Neurodegenerative Diseases (DZNE), Munich, Germany	Drafting/revision of the manuscript for content, including medical writing for content
Alle Meije Wink, PhD	Department of Radiology and Nuclear Medicine, Amsterdam University Medical Centre, Vrije Universiteit; Amsterdam Neuroscience, Brain Imaging, The Netherlands	Drafting/revision of the manuscript for content, including medical writing for content; major role in the acquisition of data
Henk Mutsaerts, MD, PhD	Amsterdam Neuroscience, Brain Imaging, The Netherlands; Ghent Institute for Functional and Metabolic Imaging (GIfMI), Ghent University, Belgium	Drafting/revision of the manuscript for content, including medical writing for content; major role in the acquisition of data
Juan Domingo Gispert, PhD	Barcelonaβeta Brain Research Center (BBRC), Pasqual Maragall Foundation, Barcelona; CIBER Bioingeniería, Biomateriales y Nanomedicina (CIBER-BBN), Madrid; IMIM (Hospital del Mar Medical Research Institute); Universitat Pompeu Fabra, Barcelona, Spain	Drafting/revision of the manuscript for content, including medical writing for content; major role in the acquisition of data
Joanna M. Wardlaw, MD	Centre for Clinical Brain Sciences, The University of Edinburgh; UK Dementia Research Institute Centre at the University of Edinburgh, United Kingdom	Drafting/revision of the manuscript for content, including medical writing for content; study concept or design
Frederik Barkhof, MD, PhD	Department of Radiology and Nuclear Medicine, Amsterdam University Medical Centre, Vrije Universiteit, The Netherlands; Institutes of Neurology and Healthcare Engineering, University College London, United Kingdom	Drafting/revision of the manuscript for content, including medical writing for content; major role in the acquisition of data; study concept or design; analysis or interpretation of data

## Glossary

AD: Alzheimer disease
CDR: Clinical Dementia Rating
cSVD: cerebral small vessel disease
EPAD: European Prevention of Alzheimer's Dementia
FLAIR: fluid-attenuated inversion recovery
FRS: Framingham risk score
PVSs: perivascular spaces
RBANS: Repeatable Battery for the Assessment of Neuropsychological Status
ROC: rates of change
SEM: structural equation modeling
VRFs: vascular risk factors
WMHs: white matter hyperintensities
